# Parliament and the Professions

**Published:** 1920-05-29

**Authors:** 


### Parliament and the Professions.

### Medical Officers in Nigeria.

It was announced that steps are being taken to revise the salaries of officers of the West African Medical Staff, and when the new salaries are settled they will be brought to the notice of possible candidates.

### Dental Caries.

Mr. Haslam, in a question to the Minister of Health, mentioned the statistics recorded by Dr. James Wheatley, County and School Medical Officer for Shropshire, that since the war there has been a very pronounced decrease in the number of children suffering from decayed teeth, the average percentage of teeth free from decay at the age of five years being recorded as 5 per cent. during the years 1910 to 1914 inclusive, and 44 per cent. during the last six months, at the age of twelve the figures being 2.9 and 27.1 respectively. Mr. Haslam suggested that the condition of children's teeth is due to the modifications of the food supply during the war, and especially to the extraction of wheat for use in bread being increased from about 68 per cent. to 80 per cent., and also to the lessened consumption of sugar. He asked if the Minister is aware that in those countries where the principal food of the people consists of cereals of high extraction, such as in the northern provinces of India, decay of the teeth is rarely met with. Dr. Addison replied that his attention had been drawn to the statement referred to, and the subject of dental caries and its prevention is receiving the careful consideration of his medical advisers.

### Sheffield Street Hospital for Women.

Mr. A. T. Davies asked the Ministry of Health whether he is aware that two men doctors have been appointed as medical officers of the Sheffield Street Hospital for Women, Clare Market, one of whom is only twenty-four years of age, and whether steps can be taken to appoint women doctors to posts of this kind. Dr. Addison said that the facts were as stated, and that he will bring to the notice of the authorities of the hospital the desirability of appointing women doctors to this kind of post.

